# Supplementary material for: Identification, Expression and Antimicrobial Functional Analysis of Interleukin-8 (IL-8) in Response to Streptococcus iniae and Flavobacterium covae in Asian Seabass (Lates calcarifer Bloch, 1790)
Source: Animals (Basel). 2024 Jan 31;14(3):475. doi: 10.3390/ani14030475 (PMC10854937; doi:10.3390/ani14030475)
Supplement: Supplementary file 1 [file animals-14-00475-s001.zip › animals-2769947-supplementary.pdf]

Supplementary material Table S1. Muangrerk *et al*. (2023)

Versus	Identity (%)	Similarity (%)
L. calcarifer VS C. elaphus	30.4	50.0
L. calcarifer VS B. taurus	30.4	50.0
L. calcarifer VS O. aries	31.4	51.0
L. calcarifer VS F. catus	36.3	52.9
L. calcarifer VS C. lupus	31.4	51.0
L. calcarifer VS S. scrofa	34.6	50.5
L. calcarifer VS O. cuniculus	35.3	52.9
L. calcarifer VS H. sapiens	34.6	52.9
L. calcarifer VS G. gallus	33.3	53.7
L. calcarifer VS M. gallopavo	34.3	53.7
L. calcarifer VS C. livia	34.0	53.8
L. calcarifer VS G. sonneratii	38.1	57.1
L. calcarifer VS A. platyrhynchos	37.7	52.8
L. calcarifer VS X. tropicalis	38.3	58.9
L. calcarifer VS C. phantasma	30.8	51.0
L. calcarifer VS T. scyllium	41.2	59.8
L. calcarifer VS D. labrax	24.8	46.0
L. calcarifer VS A. latus	76.6	86.2
L. calcarifer VS L. crocea	68.1	86.2
L. calcarifer VS K. marmoratus	69.1	82.5
L. calcarifer VS A. ocellaris	64.9	74.5
L. calcarifer VS S. salar	51.0	62.2
L. calcarifer VS O. mykiss	53.1	62.2
L. calcarifer VS E. lucius	40.4	59.6
L. calcarifer VS L. rohita	49.5	64.9
L. calcarifer VS C. carpio	49.0	62.2
L. calcarifer VS C. idella	53.5	65.7
L. calcarifer VS H. nobilis	53.5	64.6
L. calcarifer VS H. molitrix	51.5	62.6
L. calcarifer VS M. aeglefinus	44.6	67.3
L. calcarifer VS T. rubripes	46.9	63.3
L. calcarifer VS O. niloticus	46.5	63.6
L. calcarifer VS S. schlegelii	51.5	65.7
L. calcarifer VS C. striata	44.1	60.8
L. calcarifer VS S. maximus	46.5	64.6
L. calcarifer VS P. olivaceus	42.2	59.6
